# Magnetic Resonance Imaging for Rapid Screening for the Nephrotoxic and Hepatotoxic Effects of Microcystins

**DOI:** 10.3390/md11082785

**Published:** 2013-08-05

**Authors:** Aleksandra Milutinović, Ruda Zorc-Pleskovič, Marko Živin, Andrej Vovk, Igor Seša, Dušan Šuput

**Affiliations:** 1 Institute of Histology and Embryology, Faculty of Medicine, University of Ljubljana, Korytkova 2, Ljubljana 1000, Slovenia; E-Mails: sandra.milutinovic@yahoo.com(A.M.); ruda.zorc-pleskovic@mf.uni-lj.si (R.Z.-P.); 2 Institute of Pathophysiology, Faculty of Medicine, University of Ljubljana, Zaloška 4, Ljubljana 1000, Slovenia; E-Mails: marko.zivin@mf.uni-lj.si (M.Z.); andrej.vovk@mf.uni-lj.si (A.V.); 3 Centre for Clinical Physiology, Faculty of Medicine, University of Ljubljana, Vrazov trg 2, Ljubljana 1000, Slovenia; 4 Institute Jožef Stefan, Jamova 23, Ljubljana 1000, Slovenia; E-Mail: igor.sersa@ijs.si

**Keywords:** microcystin, chronic toxicity, Magnetic Resonance Imaging, kidney, liver, nephrotoxic

## Abstract

*In vivo* visualization of kidney and liver damage by Magnetic Resonance Imaging (MRI) may offer an advantage when there is a need for a simple, non-invasive and rapid method for screening of the effects of potential nephrotoxic and hepatotoxic substances in chronic experiments. Here, we used MRI for monitoring chronic intoxication with microcystins (MCs) in rat. Male adult Wistar rats were treated every other day for eight months, either with MC-LR (10 μg/kg i.p.) or MC-YR (10 μg/kg i.p.). Control groups were treated with vehicle solutions. T_1_-weighted MR-images were acquired before and at the end of the eight months experimental period. Kidney injury induced by the MCs presented with the increased intensity of T_1_-weighted MR-signal of the kidneys and liver as compared to these organs from the control animals treated for eight months, either with the vehicle solution or with saline. The intensification of the T1-weighted MR-signal correlated with the increased volume density of heavily injured tubuli (*R*^2^ = 0.77), with heavily damaged glomeruli (*R*^2^ = 0.84) and with volume density of connective tissue (*R*^2^ = 0.72). The changes in the MR signal intensity probably reflect the presence of an abundant proteinaceous material within the dilated nephrons and proliferation of the connective tissue. T_1_-weighted MRI-is a valuable method for the *in vivo* screening of kidney and liver damage in rat models of intoxication with hepatotoxic and nephrotoxic agents, such as microcystins.

## 1. Introduction

Microcystin-LR (MC-LR) and microcystin-YR (MC-YR) are toxic monocyclic heptapeptides characterized by the presence of an unusual amino acid, (all-*S*,all-*E*)-3-amino-9-methoxy-2,6,8-trimethyl-10-phenyldeca-4,6-diene acid ADDA, in their structure. They are produced by some species of cyanobacteria, found in both freshwater and in the marine environment [1,2,3,4]. Cyanobacterial blooms are becoming more frequent, probably due to eutrophication and climate change. Microcystins (MCs) have been the cause of human and animal health problems and even death of patients [5,6,7]. As MCs present a serious hazard to human health, the World Health Organization (WHO) published a provisional guideline value for MC-LR in 0.001 mg/L of drinking water [8].

Following absorption, the uptake of MCs into the cells occurs via the specific bile acid carriers. These carriers are present in several cell types, especially in liver, ileum, kidney and brain [9,10,11,12]. They inhibit serine/threonine phosphatases (PP1 and PP2A) [13,14], increase formation of reactive oxygen species (ROS), induce DNA damage [15,16], interact with mitochondrial ATP synthesis [17] and bind to aldehyde dehydrogenase [18]. Microcystin LR inhibits redox complexes, thus inhibiting oxidative phosphorylation in the mitochondria from kidney, which can result in kidney injury [19].

The acute intoxication with MCs causes necrosis and apoptosis of hepatocytes and other cells [20], autophagy [21], collapse of actin filaments in hepatocytes [22,23], disorganization of the hepatic micro-architecture, breakdown of sinusoidal structures and pooling of blood in the liver [24,25,26]. It has been shown that chronic intoxication with MCs promotes liver tumor formation [27,28], induces kidney injury [29,30] and causes atrophy and fibrosis of the heart muscle [31,32]. Carcinogenesis and cytoskeleton disruption may also be due to the decrease of transcription levels of several cytoskeletal genes [33]. Histopathological findings in animals after chronic application of MCs revealed that kidneys were more affected than liver. This indicates a possible adaptation of liver to the chronic action of MCs [30]. Kidneys revealed different stages of chronic inflammation, degeneration and dilatation of tubules filled with homogenous eosinophilic material. Epithelial tubular cells underwent ballooning degeneration, apoptosis and necrosis [30]. These changes were similar to the changes in kidneys found in experiments in rats that survived a short period after being intoxicated with a high dose of MC-LR [34]. On kidney cell line, Vero-E6, it has been shown that autophagy is the initial cellular effect of MC-LR, followed by lysosome destabilization and impaired mitochondrial morphology and function [35]. 

Use of a non-invasive technique in toxicology, such as magnetic resonance (MR) imaging, offers the advantage of providing information, whilst maintaining the integrity of the various organs in the body and their natural physiological and biochemical environment [36,37,38,39,40]. *In vivo* visualization of kidney injury may thus offer an advantage when there is a need for a non-invasive and rapid method for the screening of the effects of potential nephrotoxins in chronic experiments. 

The objective of this study is to visualize *in vivo* the degenerative changes of kidneys caused by chronic intoxication with MC-LR and MC-YR. Previous studies of the chronic intoxication with cylindrospermopsin [41] or MCs have shown proliferation of connective tissue and dilatation of tubules and glomeruli [19,29] filled with proteinaceous material. It is reasonable to assume that such changes might be detected by MRI and that the intensity of the MR signal should change significantly. In this study, MRI findings were compared to the *post-mortem* histopathological findings on kidneys obtained from the same animals. Signal intensity form T1-weighted images was correlated to the volume density of injured tubules, the volume density of connective tissue and the percentage of heavily damaged renal corpuscles. 

## 2. Results and Discussion

**Figure 1:**
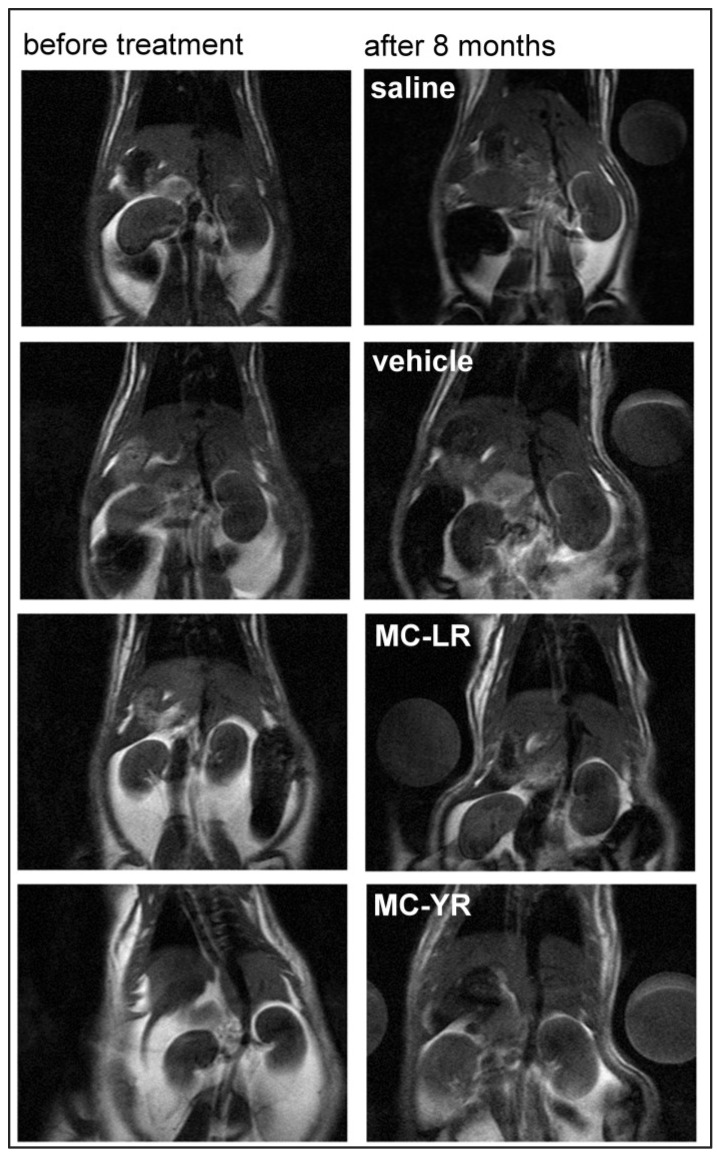
T1-weighted magnetic resonance (MR) images from rats at the beginning of experiment and after eight months of treatment. The left column shows the images of thorax and abdomen of animals at the beginning of the experiment and the right column the images from animals after treatment with physiological saline, vehicle (0.8% ethanol and 0.2% methanol dissolved in 0.9% saline), microcystin LR and microcystin YR.

MR imaging is a useful tool for the detection of pathological changes in acute and in chronic diseases and for following organ damage in clinical and biomedical research. [37,42,43]. Pathological changes after experimental intoxication with MCs were usually monitored using biopsy, an invasive *in vivo* technique, or post mortem with histopathological examinations of tissue samples. Only two studies of the effects of MCs have been described using MR imaging as a non-invasive *in vivo* method, but it was employed for the evaluation of the effects of MCs on liver [36,39]. After acute intoxication with MC-LR, the pathological changes in the liver were monitored using 1H-NMR on T2-weighted imaging using a 7T Varian scanner. The imaging revealed an increase in signal intensity proximal to the hepatic portal vein, but there is no data on the effects of chronic exposure to MCs [39]. In the second study, the T1-weighted MR imaging was used to investigate the effect of acute intoxication with cyanobacterial lyophilization rich with MCs on the liver of rabbits [36]. Histological analysis showed that changes seen on MR images represented liver injury characterized with fatty infiltration and periportal fibrosis. Electron paramagnetic resonance (EPR) and MRI studies of the acute effects of nodularin have shown that nodularin causes severe liver hypoxia and an increased T2 signal in liver tissue near the “porta hepatis”, but not in the peripheral portions of liver. Chemical shift sensitive imaging revealed periportal edema [44]. There has been no report on MRI evaluation of acute or chronic microcystin-induced kidney injury, although non-invasive *in vivo* assessment of kidney structure during chronic experiments would be useful to determine the time-course of nephrotoxicity and to select the appropriate timing for sample collection. Chronic exposure of animals to MCs affects liver [24,25,26], kidney [30,34], heart [31], brain [10,45,46] and many other organs. This makes several months-long longitudinal studies with multiple repeated anesthesias complicated to perform, because the animals’ response to anesthesia may change with the progress of the disease, leading to organ failure. There is also no report in the literature on the possible interactions between the effects of MCs and anesthetics, and in our study, multiple anesthesias were avoided for the above mentioned reasons and due to legal and ethical issues. We have scanned the animals in the beginning and at the end of the eight months’ period. We used a simple T1-weighted Multi-Slice-Multi-Echo (MSME) technique on a 2.35 T MR scanner (Figure 1) to detect the changes in the kidney parenchyma induced with purified MCs LR and YR in a chronic experiment, as the preliminary experiments showed that T1-weighed images were informative for the assessment of MC induced kidney pathology, and the scanning time was relatively short. The relative signal intensity from kidneys at the beginning of the experiment before the animals received any treatment was 1.08 ± 0.15 (mean ± SD).

At the end of the experiment, the signal intensity of T1-weighted MR images of rat kidneys, measured in coronal projection, increased significantly. The ratio between the average signal intensity of both kidneys and the signal intensity of water phantom on the same MR image was calculated. The average intensity of the T1-weighted MR signal of the kidneys of MC-LR treated rats (1.64 ± 0.17) was significantly higher compared to control groups, *i.e.*, vehicle treated (1.13 ± 0.11) and saline treated group (1.02 ± 0.02). The average signal intensity of kidneys in the MC-YR treated group was also increased, but the increase was not statistically significant (1.40 ± 0.24).

**Figure 2:**
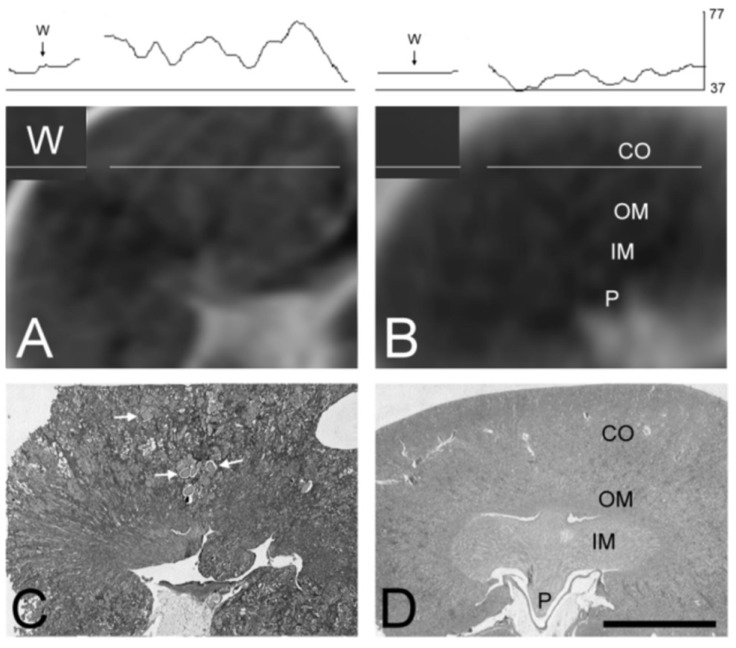
T1-weighted MR images of rat kidney compared to gross histology. The upper trace shows the signal intensity profile of water phantom and kidney parenchyma expressed in gray levels (0–255). The white lines positioned over the water phantom (W) and the kidney cortex (CO) show the position where this signal intensity-profile was acquired. Labels on the MR images correspond to kidney structure verified on the histologic sections. MR images showing kidney regions matching the histological slices are shown in A and B. MR image from the MC-LR treated animal shows more granular structure (**A**), while the MR image from the control animal is darker and more homogeneous (**B**). Paraffin sections (4 μm) stained with hematoxylin and eosin are shown as relative optical density images acquired by the black and white video camera. The slice from an animal treated with MC-LR (10 μg/kg i.p.) every other day for eight months is shown in (**C**), and the image from the control animal equivalently treated with the vehicle (0.8% ethanol and 0.2% methanol dissolved in 0.9% saline) is shown in (**D**). The kidney sample from the MC-LR treated rat shows nephropathy characterized by numerous tubular hyaline casts (**C**, white arrows). Kidney from the control rat reveals normal structure. Bar = 5 mm. P—renal papilla, IM—inner medulla, OM—outer medulla, CO—kidney cortex. Corresponding labels of the kidney structure seen on histopathological slices are also marked on MR images, where the kidney structure is less evident.

**Figure 3:**
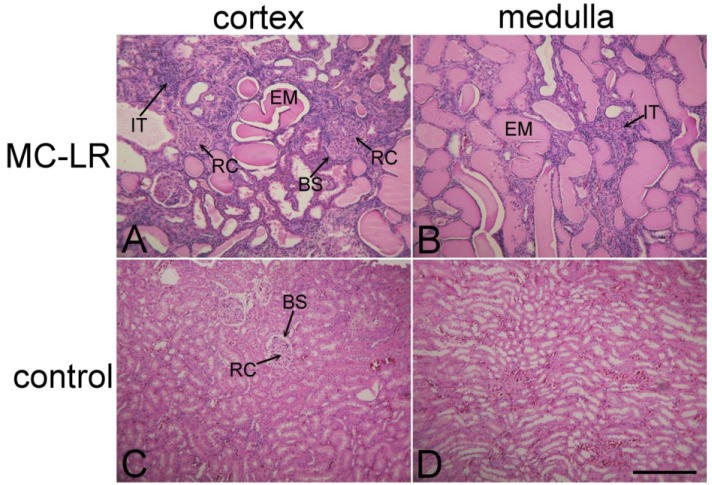
Nephrotoxic effects of microcystin LR. Kidney slices stained with hematoxylin and eosin in rat kidney cortex (**A**,**C**) and medulla (**B**,**D**). The experimental rat was treated with MC-LR (10 μg/kg i.p.) (**A**,**B**), every other day for eight months. The control rat was treated only with the vehicle (containing 0.8% ethanol, 0.2% methanol and 0.9% NaCl) (**C**,**D**). In the MC-LR treated rat, numerous enlarged renal corpuscles (RC) with compressed Bowman’s space (BS) are seen (**A**). The tubules are widened and filled with eosinophilic material (EM), as seen in (**A**,**B)**. In-growing interstitial tissue (IT) (**A**,**B**) is infiltrated with mononuclear cells. Normal histological structure of kidney from the control rat is shown in (**C**,**D)**. Bar = 300 μm.

The results show that the lesions of the kidneys in the MC-LR group after the eight months’ treatment period could be detected *in vivo* by the use of MR imaging (Figure 1 and Figure 2A,B). This is in agreement with the previous data showing that the tubuli and peritubular spaces are filled with proteinaceous material. As T1 is shortened in the presence of proteins in water solution, the increase in T1 signal intensity is in accordance with the histopathology. Kidney damage was more extensive in the MC-LR than in the MC-YR group (Figure 3). 

During the initial months of the experimental period, the MC-treated rats did not appear significantly affected by the treatment. Toward the end of the experiment, the rats treated with MC-LR and rats treated with MC-YR developed a hunched posture, reduced motor activity and reduced resistance at handling. At that time, the histopathological examination of HE stained sections of the kidney from the MCs-treated rats revealed nephropathy, as shown in Figure 2 and Figure 3. This is in good agreement with the findings of Leung *et al.* [47], who reported that the T1-weighted signal of the human kidneys was increased in patients that had glomerulonephritis with nephrotic syndrome characterized by numerous, necrotic and widened tubules jammed with eosinophilic material. Nephropathy caused by the chronic exposure to MCs (Figure 2C and Figure 3A,B) is also characterized by numerous degenerated renal corpuscles with collapsed glomeruli and widened Bowman’s space, necrotic and widened proximal and distal tubules, interstitial edema with mononuclear infiltration and moderate fibrosis. Several renal corpuscles contained collapsed tufts of glomerular capillaries, and the Bowman’s space was filled with eosinophilic material. In other renal corpuscles, the Bowman’s space was compressed (Figure 3A), and some of the renal corpuscles had a thickened Bowman’s capsule.

Kidneys in the MC-LR group appeared more affected than those in the MC-YR group in MR images and in tissue samples. The percentage of heavily injured renal corpuscles in the MC-LR group (48.41% ± 13.22%) was significantly higher compared to the MC-YR (24.39% ± 7.13%), vehicle (3.11% ± 1.55%) and saline (0.80% ± 0.57%) groups. The epithelium of the numerous convoluted tubules in MC-treated groups had flattened epithelial cells, pycnotic nuclei, increased vacuolization of the cytoplasm and the eosinophilic cytoplasm of necrotic cells. Numerous tubules in kidneys from MC-treated experimental animals were widened and filled with eosinophilic material (Figure 2C and Figure 3B). Tubular epithelial cells were often desquamated or entirely missing. Interstitial space was infiltrated by lymphocytes and appeared edematous. In-growth of the connecting tissue was also evident (Figure 3A,B).

After MR imaging, the animals were sacrificed, and histopathological examination of the kidneys was performed. In the control groups, normal kidney structure was revealed both with MR-imaging (Figure 2B) and with hematoxylin and eosin (HE) staining (Figure 3C,D). Histopathology revealed that kidneys in the MC-LR group were significantly more affected than those in MC-YR group. The data are comparable to the earlier findings reported in the study of kidney tissue injury after the chronic treatment of animals with MC-LR and MC-YR [30]. The analysis was focused on the volume densities of histological changes, which may explain the observed increase in the T1 MRI signal. The volume density of heavily injured renal tubules in the MC-LR group (0.18 ± 0.05 mm^3^/mm^3^) was significantly higher compared to the vehicle (0.06 ± 0.02 mm^3^/mm^3^) and saline group (0.03 ± 0.01 mm^3^/mm^3^). The volume density of highly injured renal tubules in the MC-YR group (0.11 ± 0.01 mm^3^/mm^3^) was significantly higher compared to the saline group. The volume density of connective tissue in the MC-LR group (0.26 ± 0.06 mm^3^/mm^3^) was significantly higher compared to the MC-YR (0.12 ± 0.07 mm^3^/mm^3^), vehicle (0.11 ± 0.05 mm^3^/mm^3^) and saline groups (0.03 ± 0.01 mm^3^/mm^3^) (Figure 3C). There was no difference in the volume density of connective tissue in the MC-YR, vehicle and saline group. 

**Figure 4:**
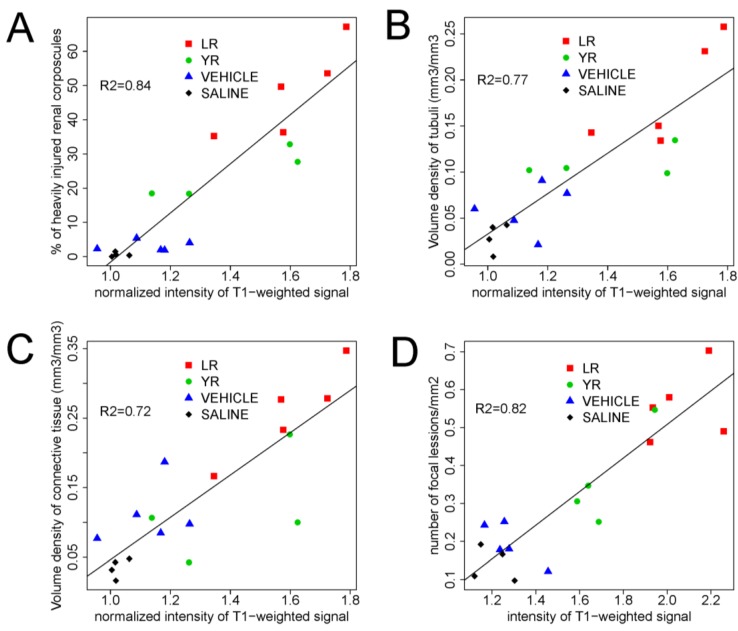
Correlation of the T_1_-weighted signal intensity with histopathological findings. There is a significant linear correlation between the intensity of T_1_-weighted MR signal (mean ± SD) and the percentage of heavily injured glomeruli (**A**), the volume density of heavily injured tubules (**B**) and the volume density of connective tissue (**C**) and the number of focal lesions in liver (**D**). Groups of animals are color-coded. Treatments: MC-LR (10 μg/kg), MC-YR (10 μg/kg), vehicle (a mixture of 0.8% ethanol, 0.2% methanol and 0.9% NaCl) and physiological saline. The injections (i.p.) were given every other day for eight months. Statistical analysis: linear regression on individual data.

Analysis showed a positive correlation between the intensity of T1-weighted signal in MR images and the extent of kidney injury assessed by the percentage of heavily injured renal corpuscles (*R*^2^ = 0.84), the percentage of heavily injured tubules (*R*^2^ = 0.77) and the fraction of connective tissue (*R*^2^ = 0.72) measured on the HE stained slices (Figure 3A–C). Signal intensity is significantly different (*p* < 0.05) between the MC-LR group and both control groups, while the MC-YR group differs significantly only from the saline control group. In liver, the average intensity of the T1-weighted MR signal in the MC-LR treated group (2.06 ± 0.15) and MC-YR group (1.71 ± 0.16) was significantly higher compared to the vehicle treated group (1.28 ± 0.11) and to the saline treated group (1.2 ± 0.09). The signal intensity increase of T1-weighed images from liver also correlated with the extent of liver injury (*R*^2^ = 0.82), as shown in Figure 4D.

This is in agreement with the findings of several authors who have found an increase in the T1-weighted signal of the human kidneys in patients with kidney inflammation, interstitial edema [47,48] and interstitial edema with early coagulation necrosis [49]. It is known that on T1-weighted images, the increased signal intensity within renal cysts may indicate the presence of hemorrhage or accumulation of fluid containing proteins [50,51,52]. Present data show that the inflammatory changes, such as the edema of connective tissue, degeneration and necrosis of nephrons and the abundant proteinaceous material within the dilated nephrons, may be responsible for the increase of the T1-weighted MR signal in the MC-LR-treated group of experimental animals. MR imaging allows *in vivo* detection of kidney pathology. It has a potential for continuous monitoring of the effects of nephrotoxic and hepatotoxic substances.

## 3. Experimental Section

### 3.1. Animals and Treatment

We used male Wistar rats weighing from 400 g to 500 g at the beginning of the experiment. The animals were handled following the guidelines in the Slovenian Law for Animal Health Protection and Instructions for Granting Permit for Animal Experimentation for Scientific Purposes. Rats were treated every other day for 8 months with MC-LR and MC-YR in relatively low doses (10 μg MC-LR/kg i.p., *n* = 5; 10 μg MC-YR/kg i.p., *n* = 4). The control group was treated with vehicle (*n* = 5) (0.8% ethanol and 0.2% methanol dissolved in 0.9% saline) in a volume of 3.7 mL/kg or pure saline (*n* = 4). MC-LR and MC-YR were isolated as described before [53,54]. The animals were anaesthetized with the i.p. injection of Rompun^®^ (2% xylazine hydrochloride, Bayer, Leverkusen, Germany; 0.65 mL/kg), Ketanes^®^ (ketamine hydrochloride; Parke Davies, Wien, Austria; 2.5 mL/kg), and atropine (Belupo, Koprivnica, Croatia; 0.3 mL/kg). At the end of experiment, the animals were sacrificed in CO_2_ anesthesia.

### 3.2. Magnetic Resonance (MR) Imaging

MR-imaging was performed on a 2.35 T Bruker Biospec system (Bruker Instruments, Germany). A T1-weighted Multi-Slice-Multi-Echo (MSME) technique was used with the following parameter settings: repetition time, TR = 400 ms, time to echo, TE = 18 ms, repetitions = 10, number of slices = 6 to 7, slice thickness = 4 mm; the field of view was quadratic, ranging from 9 × 9 to 13 × 13 cm, matrix, 256 × 256. In-plane image resolution was from 350 to 500 µm per pixel.

MR imaging was performed at the beginning and at the end of the eight months’ experimental period. A container with distilled water (water phantom) was placed next to each rat to allow the correction of the variability of the signal intensity between individual MR scans. Anaesthetized animals and the water phantom were fixed by an adhesive tape on a custom made cardboard platform. The platform was then positioned in the radio frequency coil, always at the same place, by using predetermined landmarks.

Since this study focused on anatomical changes in the kidney resulting from chronic exposure to MC, the T1-weighted Multi-Slice Spin-Echo pulse sequence, which provides excellent structural information, was selected as the best compromise between image quality (contrast) and acquisition speed. Other sequences, such as gradient echo (FLASH, SSFP), fast spin-echo (RARE) or diffusion-weighted imaging (DWI) would also provide useful information for assessment of the acute kidney injury. However, these sequences were either too demanding for the gradient hardware (RARE) or were prone to motion artefacts, due to breathing and cardiac pulsation (DWI) or to susceptibility artefacts (FLASH, SSFP). It would also be interesting to employ T1 or T2 mapping sequences, as the relaxation maps provide more accurate and comprehensive information than the corresponding T1- or T2-weighted images, but their acquisition time is usually very long, so that the methods are difficult to perform in *in vivo* animal study experiments. In addition, contrast enhanced MRI was also not included in the study, as application of contrast agents would pose an additional stress to the animals.

### 3.3. Preparation, Fixation and Staining with Hematoxylin and Eosin (HE)

The kidneys were quickly removed, fixed in buffered 10% formalin for 24 h and embedded in paraffin. Microtome sections (4 µm) were then cut and stained with HE.

### 3.4. Quantification of the Signal Intensity of T1-Weighted MR Images

The signal intensity of MR images was measured by using the UTHSCA Image Tool software (San Antonio, TX, USA). For each animal, the measurements were performed on three consecutive coronal image slices of the kidneys. On each slice, the contours of both kidneys were manually outlined. 

The signal intensity of the kidney was calculated as the ratio between the average intensity of the signal of both kidneys divided by the intensity of the water phantom signal. For each treatment group, the average signal intensity was calculated. 

### 3.5. Quantification of the Extent of the Injury of Renal Corpuscles, Renal Tubules and Connective Tissue Stained with HE

The evaluation of the extent of the injury of renal corpuscles, renal tubules and the ingrowths of the connective tissue was performed under light microscope (Leica DMIL, Wetzlar, Germany) by the trained observer, who was unaware of the treatment protocols. 

The number of renal corpuscles that were grouped in two categories were counted on three histological sections of the kidneys of each animal: (1) intact/mildly injured, as judged by a normal appearance of globular capillary tufts and urinary space or slightly widened Bowman’s space, and (2) heavily injured, showing thickened basement membrane, collapsed glomerular capillary tufts and widely enlarged Bowman’s space, filled with large amounts of eosinophil material or the final stage presented as compressed Bowman’s space with ingrowing connective tissue. For each section, the percentage of heavily injured renal corpuscles was calculated (100% = total number of renal corpuscles).

Stereological analysis [55] of volume density of the heavily injured tubules and of connective tissue was performed on three histological sections of each animal in the region of kidney cortex using Weibel’s test system. Volume density of the heavily injured tubules and of the connective tissue was estimated by counting points of the grid system that hit the observed area and the reference space at a magnification of 400×. The injury of distal and proximal tubules in the cortex of the kidney was severe, and on some of the HE-stained sections, it was virtually impossible to distinguish between the proximal and distal (convoluted) part of the tubules. Therefore, the proximal and distal convoluted tubules were analyzed together. The characteristics of the heavily injured tubules were: ingrowth of tubular epithelium, flattened epithelial cells with pycnotic nuclei, increased vacuolization of cytoplasm, swollen cells, eosinophilic cytoplasm of necrotic cells, cell shedding, eosinophil material deposited in the lumen of tubules and widened tubules. 

### 3.6. Statistical Analysis

For each group, the mean and SD of the measured parameters was calculated. The statistical significance of the differences between the means of the measured parameters of the treatment groups was evaluated by the one-way analysis of variance (ANOVA) followed by Scheffe’s *post hoc* analysis (*p* < 0.05). Linear regression analysis (least-squares method) was performed (*R*^2^) to determine the correlation between the percentage of heavily injured renal corpuscles, volume density of heavily inured tubules, volume density of connective tissue and the intensity of T1-weighted signal.

## 4. Conclusions

T1-weighted MR imaging is a well-suited technique for the *in vivo* detection and evaluation of kidney injury induced by chronic exposure to relatively low doses of MCs and possibly other toxins that affect kidney.

There is a good correlation between the extent of kidney injury and the intensity of the T1-weighted MR signal. This can be used as an estimate of the progression of the MC-induced kidney degeneration.

## References

[1] Dawson R.M. (1998). The toxicology of microcystins. Toxicon.

[2] Carmichael W.W., Beasley V., Bunner D.L., Eloff J.N., Falconer I., Gorham P., Harada K., Krishnamurthy T., Yu M.J., Moore R.E. (1988). Naming of cyclic heptapeptide toxins of cyanobacteria (blue-green algae). Toxicon.

[3] Blunt J.W., Copp B.R., Keyzers R.A., Munro M.H., Prinsep M.R. (2013). Marine natural products. Nat. Prod. Rep..

[4] Zurawell R.W., Chen H., Burke J.M., Prepas E.E. (2005). Hepatotoxic cyanobacteria: A review of the biological importance of microcystins in freshwater environments. J. Toxicol. Environ. Health B Crit. Rev..

[5] Soares R.M., Yuan M., Servaites J.C., Delgado A., Magalhaes V.F., Hilborn E.D., Carmichael W.W., Azevedo S.M. (2006). Sublethal exposure from microcystins to renal insufficiency patients in Rio de Janeiro, Brazil. Environ. Toxicol..

[6] Jochimsen E.M., Carmichael W.W., An J.S., Cardo D.M., Cookson S.T., Holmes C.E., Antunes M.B., de Melo Filho D.A., Lyra T.M., Barreto V.S. (1998). Liver failure and death after exposure to microcystins at a hemodialysis center in Brazil. N. Engl. J. Med..

[7] Pouria S., de Andrade A., Barbosa J., Cavalcanti R.L., Barreto V.T., Ward C.J., Preiser W., Poon G.K., Neild G.H., Codd G.A. (1998). Fatal microcystin intoxication in haemodialysis unit in Caruaru, Brazil. Lancet.

[8] (2011). Guidelines for Drinking Water Quality Toxic Cyanobacteria.

[9] Eriksson J.E., Gronberg L., Nygard S., Slotte J.P., Meriluoto J.A. (1025). Hepatocellular uptake of 3H-dihydromicrocystin-LR, a cyclic peptide toxin. Biochim. Biophys. Acta.

[10] Fischer W.J., Altheimer S., Cattori V., Meier P.J., Dietrich D.R., Hagenbuch B. (2005). Organic anion transporting polypeptides expressed in liver and brain mediate uptake of microcystin. Toxicol. Appl. Pharmacol..

[11] Hooser S.B., Kuhlenschmidt M.S., Dahlem A.M., Beasley V.R., Carmichael W.W., Haschek W.M. (1991). Uptake and subcellular localization of tritiated dihydro-microcystin-LR in rat liver. Toxicon.

[12] Runnegar M.T., Gerdes R.G., Falconer I.R. (1991). The uptake of the cyanobacterial hepatotoxin microcystin by isolated rat hepatocytes. Toxicon.

[13] MacKintosh C., Beattie K.A., Klumpp S., Cohen P., Codd G.A. (1990). Cyanobacterial microcystin-LR is a potent and specific inhibitor of protein phosphatases 1 and 2A from both mammals and higher plants. FEBS Lett..

[14] Sun Y., Zheng Q., Sun Y.T., Huang P., Guo Z.L., Xu L.H. (2013). Microcystin-LR induces protein phosphatase 2A alteration in a human liver cell line. Environ. Toxicol..

[15] Ding W.X., Shen H.M., Ong C.N. (2000). Critical role of reactive oxygen species and mitochondrial permeability transition in microcystin-induced rapid apoptosis in rat hepatocytes. Hepatology.

[16] Zegura B., Sedmak B., Filipic M. (2003). Microcystin-LR induces oxidative DNA damage in human hepatoma cell line HepG2. Toxicon.

[17] Mikhailov A., Harmala-Brasken A.S., Hellman J., Meriluoto J., Eriksson J.E. (2003). Identification of ATP-synthase as a novel intracellular target for microcystin-LR. Chem. Biol. Interact..

[18] Chen T., Cui J., Liang Y., Xin X., Owen Young D., Chen C., Shen P. (2006). Identification of human liver mitochondrial aldehyde dehydrogenase as a potential target for microcystin-LR. Toxicology.

[19] La-Salete R., Oliveira M.M., Palmeira C.A., Almeida J., Peixoto F.P. (2008). Mitochondria a key role in microcystin-LR kidney intoxication. J. Appl. Toxicol..

[20] Zhang H., Cai C., Wu Y., Shao D., Ye B., Zhang Y., Liu J., Wang J., Jia X. (2013). Mitochondrial and endoplasmic reticulum pathways involved in microcystin-LR-induced apoptosis of the testes of male frog (*Rana nigromaculata*) *in vivo*. J. Hazard. Mater..

[21] Menezes C., Alverca E., Dias E., Sam-Bento F., Pereira P. (2013). Involvement of endoplasmic reticulum and autophagy in microcystin-LR toxicity in Vero-E6 and HepG2 cell lines. Toxicol. Vitro.

[22] Batista T., de Sousa G., Suput J.S., Rahmani R., Suput D. (2003). Microcystin-LR causes the collapse of actin filaments in primary human hepatocytes. Aquat. Toxicol..

[23] Eriksson J.E., Paatero G.I., Meriluoto J.A., Codd G.A., Kass G.E., Nicotera P., Orrenius S. (1989). Rapid microfilament reorganization induced in isolated rat hepatocytes by microcystin-LR, a cyclic peptide toxin. Exp. Cell Res..

[24] Runnegar M.T., Falconer I.R., Silver J. (1981). Deformation of isolated rat hepatocytes by a peptide hepatotoxin from the blue-green alga microcystis aeruginosa. Naunyn Schmiedebergs Arch. Pharmacol..

[25] Eriksson J.E., Toivola D., Meriluoto J.A., Karaki H., Han Y.G., Hartshorne D. (1990). Hepatocyte deformation induced by cyanobacterial toxins reflects inhibition of protein phosphatases. Biochem. Biophys. Res. Commun..

[26] Theiss W.C., Carmichael W.W., Wyman J., Bruner R. (1988). Blood pressure and hepatocellular effects of the cyclic heptapeptide toxin produced by the freshwater cyanobacterium (blue-green alga) *Microcystis aeruginosa* strain PCC-7820. Toxicon.

[27] Nishiwaki-Matsushima R., Nishiwaki S., Ohta T., Yoshizawa S., Suganuma M., Harada K., Watanabe M.F., Fujiki H. (1991). Structure-function relationships of microcystins, liver tumor promoters, in interaction with protein phosphatas. Jpn. J. Cancer Res..

[28] Sekijima M., Tsutsumi T., Yoshida T., Harada T., Tashiro F., Chen G., Yu S.Z., Ueno Y. (1999). Enhancement of glutathione *S*-transferase placental-form positive liver cell foci development by microcystin-LR in aflatoxin B1-initiated rats. Carcinogenesis.

[29] Milutinovic A., Sedmak B., Horvat-Znidarsic I., Suput D. (2002). Renal injuries induced by chronic intoxication with microcystins. Cell. Mol. Biol. Lett..

[30] Milutinovic A., Zivin M., Zorc-Pleskovic R., Sedmak B., Suput D. (2003). Nephrotoxic effects of chronic administration of microcystins-LR and -YR. Toxicon.

[31] Milutinovic A., Zorc-Pleskovic R., Petrovic D., Zorc M., Suput D. (2006). Microcystin-LR induces alterations in heart muscle. Folia Biol. (Praha).

[32] Suput D., Zorc-Pleskovic R., Petrovic D., Milutinovic A. (2010). Cardiotoxic injury caused by chronic administration of microcystin-YR. Folia Biol. (Praha).

[33] Hao L., Xie P., Li H., Li G., Xiong Q., Wang Q., Qiu T., Liu Y. (2010). Transcriptional alteration of cytoskeletal genes induced by microcystins in three organs of rats. Toxicon.

[34] Hooser S.B., Beasley V.R., Lovell R.A., Carmichael W.W., Haschek W.M. (1989). Toxicity of microcystin LR, a cyclic heptapeptide hepatotoxin from *Microcystis aeruginosa*, to rats and mice. Vet. Pathol..

[35] Alverca E., Andrade M., Dias E., Sam Bento F., Batoreu M.C., Jordan P., Silva M.J., Pereira P. (2009). Morphological and ultrastructural effects of microcystin-LR from *Microcystis aeruginosa* extract on a kidney cell line. Toxicon.

[36] Frangez R., Kosec M., Sedmak B., Beravs K., Demsar F., Juntes P., Pogacnik M., Suput D. (2000). Subchronic liver injuries caused by microcystins. Pflugers Arch..

[37] Artunc F., Rossi C., Boss A. (2011). MRI to assess renal structure and function. Curr. Opin. Nephrol. Hypertens..

[38] Foley L.M., Towner R.A., Painter D.M. (1526). *In vivo* image-guided (1)H-magnetic resonance spectroscopy of the serial development of hepatocarcinogenesis in an experimental animal model. Biochim. Biophys. Acta.

[39] Sturgeon S.A., Towner R.A. (1454). *In vivo* assessment of microcystin-LR-induced hepatotoxicity in the rat using proton nuclear magnetic resonance (1H-NMR) imaging. Biochim. Biophys. Acta.

[40] Demsar F., Roberts T.P., Schwickert H.C., Shames D.M., van Dijke C.F., Mann J.S., Saeed M., Brasch R.C. (1997). A MRI spatial mapping technique for microvascular permeability and tissue blood volume based on macromolecular contrast agent distribution. Magn. Reson. Med..

[41] Chernoff N., Rogers E.H., Zehr R.D., Gage M.I., Malarkey D.E., Bradfield C.A., Liu Y., Schmid J.E., Jaskot R.H., Richards J.H. (2011). Toxicity and recovery in the pregnant mouse after gestational exposure to the cyanobacterial toxin, cylindrospermopsin. J. Appl. Toxicol..

[42] Brown A.T., Ou X., James L.P., Jambhekar K., Pandey T., McCullough S., Chaudhuri S., Borrelli M.J. (2012). Correlation of MRI findings to histology of acetaminophen toxicity in the mouse. Magn. Reson. Imaging.

[43] Kowalczuk J., Tritt-Goc J. (2011). A possible application of magnetic resonance imaging for pharmaceutical research. Eur. J. Pharm. Sci..

[44] Towner R.A., Sturgeon S.A., Khan N., Hou H., Swartz H.M. (2002). *In vivo* assessment of nodularin-induced hepatotoxicity in the rat using magnetic resonance techniques (MRI, MRS and EPR oximetry). Chem. Biol. Interact..

[45] Feurstein D., Kleinteich J., Heussner A.H., Stemmer K., Dietrich D.R. (2010). Investigation of microcystin congener-dependent uptake into primary murine neurons. Environ. Health Perspect..

[46] Li G., Yan W., Cai F., Li C., Chen N., Wang J. (2012). Spatial learning and memory impairment and pathological change in rats induced by acute exposure to microcystin-LR. Environ. Toxicol..

[47] Leung A.W., Bydder G.M., Steiner R.E., Bryant D.J., Young I.R. (1984). Magnetic resonance imaging of the kidneys. Am. J. Roentgenol..

[48] Yamashita Y., Miyazaki T., Ishii A., Watanabe O., Takahashi M. (1995). Multilocular cystic renal cell carcinoma presenting as a solid mass: Radiologic evaluation. Abodom. Imaging.

[49] Choo S.W., Kim S.H., Jeong Y.G., Shin Y.M., Kim J.S., Han M.C. (1997). MR imaging of segmental renal infarction: An experimental study. Clin. Radiol..

[50] Mosetti M.A., Leonardou P., Motohara T., Kanematsu M., Armao D., Semelka R.C. (2003). Autosomal dominant polycystic kidney disease: MR imaging evaluation using current techniques. J. Magn. Reson. Imaging.

[51] Pedrosa I., Sun M.R., Spencer M., Genega E.M., Olumi A.F., Dewolf W.C., Rofsky N.M. (2008). MR imaging of renal masses: Correlation with findings at surgery and pathologic analysis. Radiographics.

[52] Grantham J.J., Mulamalla S., Grantham C.J., Wallace D.P., Cook L.T., Wetzel L.H., Fields T.A., Bae K.T. (2012). Detected renal cysts are tips of the iceberg in adults with ADPKD. Clin. J. Am. Soc. Nephrol..

[53] Sedmak B., Kosi G. (1997). Microcystins in Slovene freshwaters (central Europe)—First report. Nat. Toxins.

[54] Sedmak B., Elersek T. (2005). Microcystins induce morphological and physiological changes in selected representative phytoplanktons. Microb. Ecol..

[55] Weibel E.R. (1981). Stereological methods in cell biology: Where are we—where are we going?. J. Histochem. Cytochem..

